# Experimental Evidence Shows the Importance of Behavioural Plasticity and Body Size under Competition in Waterfowl

**DOI:** 10.1371/journal.pone.0164606

**Published:** 2016-10-11

**Authors:** Yong Zhang, Herbert H. T. Prins, Martijn Versluijs, Rick Wessels, Lei Cao, Willem Frederik de Boer

**Affiliations:** 1 Co-Innovation Center for Sustainable Forestry in Southern China, College of Biology and the Environment, Nanjing Forestry University, Nanjing, Jiangsu, China; 2 Resource Ecology Group, Wageningen University, Wageningen, The Netherlands; 3 State Key Laboratory of Urban and Regional Ecology, Research Center for Eco-Environmental Science, Chinese Academic of Sciences Beijing, China; Chinese Academy of Science, CHINA

## Abstract

When differently sized species feed on the same resources, interference competition may occur, which may negatively affect their food intake rate. It is expected that competition between species also alters behaviour and feeding patch selection. To assess these changes in behaviour and patch selection, we applied an experimental approach using captive birds of three differently sized Anatidae species: wigeon (*Anas penelope*) (~600 g), swan goose (*Anser cygnoides*) (~2700 g) and bean goose (*Anser fabalis*) (~3200 g). We quantified the functional response for each species and then recorded their behaviour and patch selection with and without potential competitors, using different species combinations. Our results showed that all three species acquired the highest nitrogen intake at relatively tall swards (6, 9 cm) when foraging in single species flocks in the functional response experiment. Goose species were offered foraging patches differing in sward height with and without competitors, and we tested for the effect of competition on foraging behaviour. The mean percentage of time spent feeding and being vigilant did not change under competition for all species. However, all species utilized strategies that increased their peck rate on patches across different sward heights, resulting in the same instantaneous and nitrogen intake rate. Our results suggest that variation in peck rate over different swards height permits Anatidae herbivores to compensate for the loss of intake under competition, illustrating the importance of behavioural plasticity in heterogeneous environments when competing with other species for resources.

## Introduction

Selection of feeding patches is an important process in the spatial distribution of herbivores [1]. In this paper, we experimentally test how interference competition affect intake rate, feeding behaviour and patch selection. To predict patch selection, a thorough understanding of the functional response of the herbivores is required. A functional response describes how the instantaneous intake rate changes with increasing food availability [2]. Functional responses of grazing species that differ in body size are bound to be different, and understanding these allometric relationships are required to understand when competition or facilitation occur [3,4]. A Type I functional response describes a linear increase of food intake with food availability. However, a Type II functional response, which describes that instantaneous intake rate increases asymptotically with food availability, is found for most grazing herbivores [5,6]. Geese and ducks sometimes display a Type IV functional response [7,8], which is a dome-shaped curve with a maximal intake rate at intermediate biomass densities.

In general, herbivorous Anatidae require a relatively high intake of dietary nitrogen [9,10] because of their inefficiency in converting proteins from plant food into their own body tissue and their high defecation rate. This is due to differences in amino acid profiles from plant proteins and animals proteins [11,12] and low digestibility of their food [13]. Nitrogen availability of both grasses and sedges generally decreases with increasing swards height [13,14]. This has been formulated as the forage maturation hypothesis, which states that nitrogen content declines during the development of plants while the total fibre content increases [15,16]. Moreover, a hump-shaped relationship was also found between nitrogen content and plants development [17]. Herbivorous Anatidae therefore face a trade-off between food quantity and quality [18,19]. It is expected that herbivores select patches with intermediate plant biomass (for their size), which allows them to maximize their net intake rate of digestible nutrients [20,21].

Body size is an important factor that affects the intake rate of herbivorous Anatidae and therefore also patch selection. Intake rate of herbivorous Anatidae species is determined by both peck rate and peck size. At lower sward heights, peck size decreases [5,7], and to compensate for the loss in intake rate caused by decreasing peck sizes, smaller Anatidae species generally increase their peck rate [22]. Animals can adjust their behaviour in response to a changing environment, which is described as behavioural plasticity [23]. However, when swards become too short, peck size will decrease to a level where a higher peck rate cannot completely compensate, and consequently intake rate decreases [5,24]. Larger species are unable to achieve their nitrogen requirements on short swards, and due to their larger bills they have a limited ability to compensate for the smaller peck sizes in short sward by increasing their peck rate [7,25]. However, they are able to tolerate swards with lower nitrogen values [1], which allows them to select taller, less nitrogen-rich swards, but with a larger peck size [5].

Interference competition is another important factor in determining the patch selection of herbivores [26–31], associated with the body size [32]. According to Wiens [28] the necessary conditions of interspecific competition are: (1) species must rely on the same resource and (2) joint exploitation of those resources and/or interference interactions concerning resources negatively affect either one or both species. Interference competition usually incorporates a social component, some individuals being denied access to resources by the (often aggressive) actions of others [30]. Through social interactions and aggressive encounters between competitors, the larger species may control the best patches whereas smaller species are forced to sub-optimal habitat where they experience a reduction in intake rate [33,34]. Besides, when animal spent more time on interactions, less time can be spent on foraging and therefore foraging success decreases [34–36]. Moreover, social and aggressive interactions may be very costly in terms of time and energy [37]. Thus, under the influences of interference competition intake rate may decrease. Kristiansen and Jarrett [26] examined competition between moulting Canada geese (*Branta canadensis*) and Greenland white-fronted geese (*Anser albifrons flavirostris*) by field observations in Western-Greenland. They found that Greenland white-fronted geese, the subordinate species, shifted to sub-optimal feeding patches with lower food quality and increased their foraging time. The authors predicted that they could compensate for the lower energy intake in this way. Madsen [38] found that flock size may affect the competition outcome when they studied competition between Pink-footed geese (*Anser brachyrhynchus*) and Greylag geese (*Anser anser*) in the field, as the species with a larger group size was often the dominate species within mixed flocks in the field [38,39]. Hence, interference competition is an important mechanism that determines differences in feeding patch selection in competing species.

The aim of this study is to examine the effects of interference competition of differently sized grazing Anatidae species on their feeding patch selection and foraging behaviour. For this purpose, we used an experimental approach where we offered heterogeneously distributed feeding patches with different sward heights to captive birds. These sward heights were differentially maintained through mowing. Firstly, in order to better understand the effect of interspecific competition, we quantified the functional response for each species. Then, a competition experiment was conducted with a free patch choice experiment as control. We expected that larger species are competitively superior and are expected to exclude smaller species from preferred food patches. Both superior and subordinate grazing Anatidae species are expected to increase their peck rate and/or percentage of feeding time under interference competition to compensate for the potential loss of intake rate.

## Materials and Methods

### Ethics statement

The animals used in this experiment were tame, not obtained from wild catches, but acquired from a special animal farm which complied with Chinese laws. After the experiment, all animals were returned to the farm. Field permit of this research was granted by the Shengjin Lake National Reserve, Anhui Province, China. This study was approved by the Animal ethics committee University of Science and Technology of China (Approval number: USTCACUC1205052). The experiments complied with the “Guidelines for the Treatment of Animals in Behavioural Research and Teaching”. The experimental treatments did not lead to suffering or injuries, and standard housing care was provided over the entire experimental period.

### Experimental trials

This experiment was carried out at the Shengjin Lake National Reserve in Eastern-China from 16 December 2012 to 27 February 2013. Individuals of three differently sized Anatidae species were used: wigeon (*Anas penelope*) (mean body mass of all individuals: 570 g; N = 7), swan goose (*Anser cygnoides*) (2,740 g; N = 7) and bean goose (*Anser fabalis*) (3,170 g; N = 7). However, in the field, body weight of swan goose tends to be larger than that of bean goose. We used tame birds as they were accustomed to being handled by humans, thus reducing stress. One week before the experiment, the three species were allowed to feed on nearby grassland that had a similar grass structure as the experimental area. The animals were kept in a holding pen inside a covered enclosure. The enclosure consisted of a pond where the animals could swim, eight experimental enclosures (5 × 5 m, with 2 m high wire fence) where the sedge *Carex heterolepis* was the dominant species, and four holding pens where clean water was always available (S1a and S1b Fig). All birds were individually identifiable by using different combinations of plastic tags on the legs, and before each observation the observer recorded the ID of the bird. For the functional response experiment, the swards were mowed to four particular heights representing the natural conditions at our field sites at which geese feed (1, 3, 6, 9 cm) within the 5 × 5 m enclosures. For the free choice and competition experiment, the vegetation within the enclosures was divided into sixteen 1 × 1 m patches with small paths in between so that we were able to mow the plots to their desired height without trampling the vegetation in the patches. In each row of four patches, the sward was mowed to four different sward heights of 1, 3, 6, and 9 cm following a randomized Latin square design to eliminate the effects of environmental gradients within plots (S1c Fig). Clean fresh water was provided at four different points in the enclosure. After each trial we raked the swards and measured the sward height. If the sward height was reduced, we started the observations in another exclosure. All observations were made inside a cabin at a distance of 40 m using a 18–60 × telescope (Carl Zeiss, Germany). Behavioural activities and patch selection were recorded using the software ObserverXT v10 installed on a Psion Workabout hand-held computer (Noldus Information Technology, Netherlands)

### Vegetation

**Fig 1:**
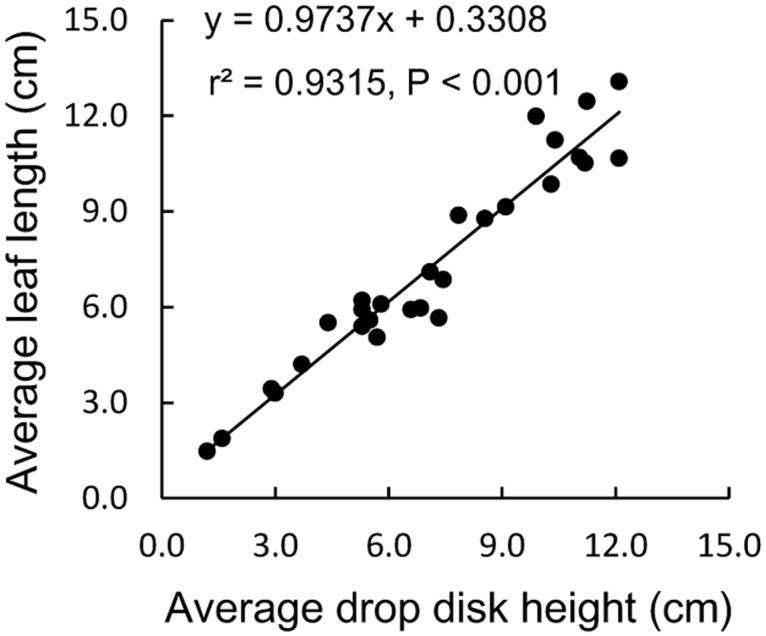
The relationship between drop disk height (cm) and leaf length (cm).

Sward height was determined for each of the 1 × 1 m patches on the day before we started with each repetition of the free choice and competition experiment. Sward height was estimated as the mean of 17 measurements taken according to a fixed pattern (once in the middle, and four on each of the 2 perpendicular lines crossing the centre and the 2 diagonals) using a drop disc meter (DPM: diameter: 10 cm, weight: 5 g) on a graduated stick, to the nearest 0.5 cm [40]. For each enclosure, the average sward height was calculated. However, in winter, sedges tend to lie flat while the tips of the leaves died off so that the leaves did not supported the weight of the drop disc. In order to correct for this effect 30 additional samples were taken and both sward height (DPM value) and leaf length (maximum length of the leaf to the soil surface) were measured by a ruler to the nearest 0.1 cm. The equation of the best-fitted line was used to adjust the measured disc pasture heights to measured leaf lengths (Fig 1). All reported heights were accordingly corrected and reported as sward heights.

We measured biomass by clipping 15 squares (20 × 20 cm) to soil level for each of the height treatments. All samples were then oven-dried at 70°C for 48 h to determine dry weight. Dry weight was measured to the nearest 0.001 g. From each biomass sample, a mixed subsample (~8 g) was taken for the analysis of the nitrogen content [41].

### Functional response experiment

The experiments were conducted from 08:00 to 12:00h. All trials were conducted after the animals had been fasting during the previous night. During the experiment, six birds from one species were put in the experimental enclosure for 1 h with a uniform grass height at a selected height (1, 3, 6, or 9 cm, respectively). One observer recorded the behaviour for a period of two minutes using focal sampling over two behavioural classes: feeding and non-feeding behaviour. A bird was considered to be feeding when it stood with its bill pointed towards the vegetation [42]. When the observation started, we recorded their behaviour at a time resolution of 1 s. When an observation ended, a next focal bird was chosen randomly from the group but excluding birds from which behavioural observations had already been collected. When all individuals had been observed we started over again with all individuals in the group under observation, thus each individual was measured 3–5 times within a trial.

The peck rate was calculated by a separate observer by measuring the time it took for a bird to take 10 to 50 pecks using a stop watch [24]. The peck rate was only measured for grazing individuals. All individuals were measured multiple times (8–12) within 1 h.

To calculate instantaneous intake rate and nitrogen intake rate, all droppings in this functional response experiment were collected in a paper bag after the observations of each species, and the animals were kept from foraging for at least six hours by putting them in containers with a mesh floor (S1d Fig). All droppings in the container were also collected [5]. Droppings were dried at 70°C for 48 h.

In one day, all three species were tested, each with six birds in the enclosure. This procedure was repeated three times on three consecutive mornings. Hereafter, we cut the grass to one of the other heights. The grass was cut from the original taller grass heights to 9, 6, 3 or 1 cm and for each particular sward height we carried out our experiments with the same procedure. To facilitate the analysis of the competition effect, the order of the species in the experiment was fixed; we first tested wigeon followed by bean goose and finally swan goose. The time of day, i.e. session number, was included as random factor in our analyses to accommodate for time-effects.

Dry weight (DW), ash free dry weight (AFDW), nitrogen and acid detergent fibre (ADF) content of vegetation samples and droppings were determined at the laboratory of Wageningen University. The ADF and DW were used to estimate the digestibility of the forage through the following calculation [43–45]:

$$Digestibility(\%AFDW)=(1-\frac{ADF_{sedge}}{ADF_{droppings}})\times 100\%$$

$$Dry\;matter\;intake=\frac{Dry\;weight\;droppings\;(g\;AFDW)}{\left(1-Digestibility\right)}$$

The instantaneous dry matter intake rate (mg/min) per animal was calculated from the total dry matter intake divided by total feeding time of all birds. The instantaneous nitrogen intake rate (mg m^-1^) was calculated by multiplying the instantaneous dry matter intake rate with the nitrogen content.

### Free choice experiment

In this experiment six individuals of one species moved freely inside the 5 × 5 m enclosure where multiple sward heights were made available. The animals were thus able to select a particular sward height for foraging without the other two species being present. The experiments were conducted from 08:00 to 11:30 h over three consecutive days, each day measuring a different species. All trials were conducted after the birds had been fasting during the previous night. This three-day series was repeated four times. The following behavioural activities were recorded for the selected bird in a 3-minute period: feeding, walking, vigilant, drinking, sitting/sleeping, social and aggressive interactions. Peck rate was recorded as described above. The order of the species was bean goose, swan goose and wigeon.

### Competition experiment

The trials were conducted between 09:00–11:30 h inside the 5 × 5 m enclosure on consecutive days, with one day in between two consecutive sessions. Every day we tested one combination of species, a standardized total group size of six animals was used (Table 1) to avoid group size effect as goose behaviour may be affected by the number of birds in a group [38,39]. The animals were randomly selected from the seven birds available per species. Each session was repeated four times. All trials were conducted after a fasting period of one night.

**Table 1:** All species combinations used in the inter-specific competition experiment. n = number of individuals participating within the combination.

Combination	Species 1	n	Species 2	n	Species 3	n
1	Wigeon	3	Bean Goose	3		
2	Wigeon	3	Swan Goose	3		
3	Bean Goose	3	Swan Goose	3		
4	Wigeon	2	Swan Goose	2	Bean Goose	2

When the trial started, all six birds were allowed to enter the enclosure and grazed freely among the patches. The 3-minute focal observations were used as above. The only difference was that aggressive interactions between individuals of the different species were also recorded, and when aggressive interactions occurred, we recorded if the focal bird was the winner or loser. This way we could also determine the interspecific dominance status of the birds. Peck rate of each animal was also recorded as described above.

### Statistical analysis

#### Vegetation

A One-Way ANOVA was used to examine differences in sward height and nitrogen content between treatments, followed by Post hoc Tukey tests to identify differences among groups.

#### Functional response

A One-Way ANOVA and Post-hoc Tukey-test was used to identify differences in percentage foraging time, peck rate, instantaneous intake rate (mg min^-1^), nitrogen intake (mg min^-1^) and peck size for different species foraging on different swards heights.

In winter, the sedge growth rate was very low and the vegetation was short. Hence, for larger animals, intake was limited by sward height. So a linear regression model was fitted to test for a Type I functional response. With increasing sward height (Height, cm), intake rate (Intake, mg min^-1^) might reach a maximal value and even decrease. So, to model nitrogen intake rate (mg min^-1^) as a function of sward heights, two candidate non-linear regression models were also fitted. To test for a Type II functional response, Holling’s model [46] was used, and for a Type IV functional response, a quadratic equation was used [5]. These three functional responses, differing in complexity from a linear relation with sward height to a dome-shaped relation, were compared using the Akaike’s Information Criterion for small sample size (AIC_C_) for model selection.

Intake=a×Height+b  (Type I)

$$Intake=\frac{a\times Height}{1+b\times Height}\;\left(\text{Type II}\right)$$

$$Intake=a+b\times Height-c\times Height^{2}\;\left(\text{Type IV}\right)$$

Heuermann et al. [7] used a Michaelis-Menten model to test the Type II functional response, and a predator confusion model for the Type IV functional response. In this research, these two models were also applied, but they poorly fitted the data and are therefore not reported. We also fitted the models using the sward biomass as independent variable. By examining the R^2^ value, sward height better fitted our data, so sward height was used in all final models.

#### Competition experiment

Individual differences may play a role in our data analysis [5], hence we first detected if the behaviour of each species differed among different individuals using the data collected from the free choice experiment. A Kruskal-Wallis test was used to test the differences of percentage of feeding time among different individuals and an *F* test was used to test for differences in peck rate among the different individual after being square root transformed.

Per species, we tested if the three differently sized competitor species affected the percentage of time foraging and being vigilant of the focal species using a General Linear Mixed Model (GLMM). The percentage foraging and vigilance was arcsin transformed to satisfy the assumptions of normality. The GLMM was conducted with competitor and sward height as fixed factors. Session, individual (accounting for individual differences) and enclosure number were always included as random factors in all models (including the null models). Within the model, we were especially interested in the interaction effect between sward height and competitor, and this interaction term was therefore kept as a fixed effect in our models. Reported models were all significantly different from the null model with only an intercept. Tukey post-hoc tests were used to identify differences in percentage foraging time over the different treatment combinations.

To determine the differences in peck rate among the three species, a GLMM was also used. Peck rate was square root transformed to satisfy the assumptions of normality. Sward height, competitor and the interaction term were included as fixed factors; session, individual (accounting for individual differences) and enclosure number were used as random factors. Tukey post-hoc tests were used to identify differences in peck rate over the different treatment combinations. All candidate models, including a null model with only an intercept, were tested and ranked in terms of △AIC.

We also calculated the mean instantaneous intake rate and the nitrogen intake for each species when foraging alone (results from the functional response experiments) and in combinations with different competitors. One-way ANOVAs were carried out to test whether the instantaneous intake rate and the nitrogen intake differed over the different species combinations. All statistical analyses were conducted in SPSS v19 (IBM Corp, 2010) and R 2.13.0 with the package lme4 and MuMin (R Development Core Team, 2012).

## Results

### Vegetation

**Fig 2:**
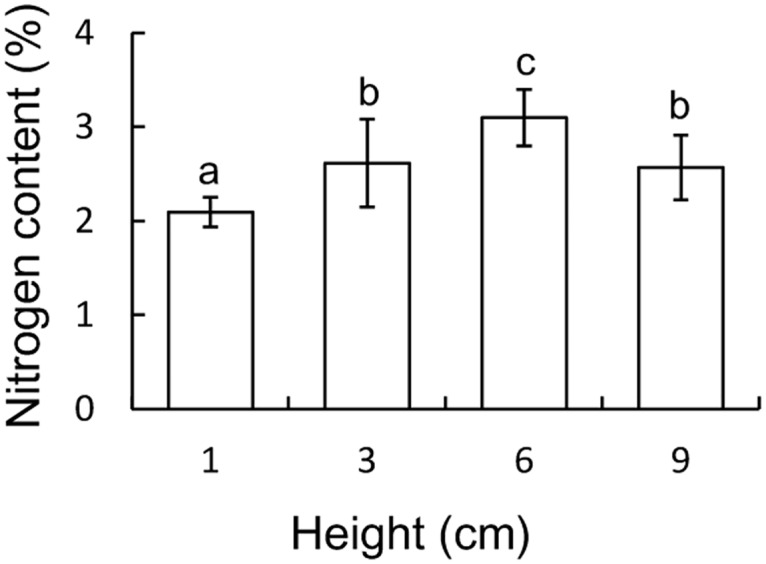
Mean values (± 95% CI) of the percentage nitrogen content within the four different sward heights. Letters indicate significant differences in nitrogen content between sward heights on the basis of a Tukey post-hoc test.

Nitrogen content differed between the four height treatments, as the 1 cm high swards, contained significantly less nitrogen than the taller swards and the highest concentration of nitrogen was recorded in the 6 cm high swards (ANOVA, *F*_3,59_ = 22.43, *p* < 0.001; Fig 2).

### Functional response experiment

**Fig 3:**
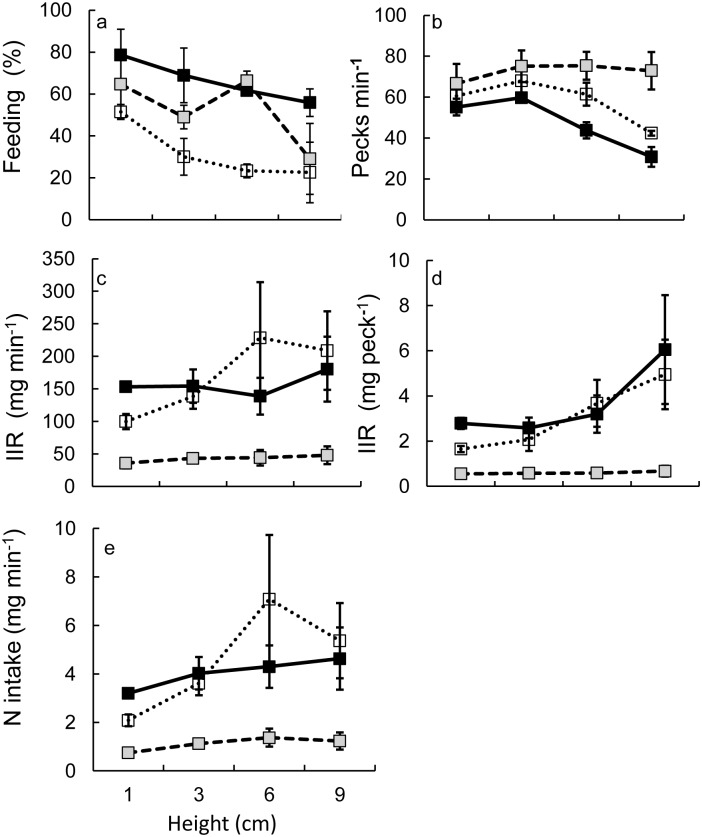
The relationship between sward height and feeding percentage (a), peck rate (b), instantaneous intake rate (IIR)(c and d) and nitrogen intake (e). Dark filled squares: swan goose; open squares: bean goose; grey filled squares: wigeon. The error bars show the ± 95% CI.

The feeding percentage of swan goose differed significantly over the different swards heights (ANOVA, *F*_3,8_ = 4.608, *p* = 0.037; Fig 3a), and the percentage feeding on 1 cm swards was significant higher than on 9 cm swards. Bean goose had a similar trend as swan goose (*F*_3,8_ = 17.39, *p* = 0.001), and percentage feeding on 1 cm swards was significantly higher than on other sward heights. Wigeon had the highest percentage feeding on 6 cm (*F*_3,8_ = 8.594, *p* = 0.007); the percentage feeding on 9 cm swards was significantly lower than on other sward heights.

The highest peck rate of swan goose was found on shorter swards (ANOVA, *F*_3,8_ = 42.66, *p* < 0.001; Fig 3b). Peck rate of bean goose also differed over the four different heights (*F*_3,8_ = 12.84, *p* = 0.002) and the peck rate on 9 cm swards was significantly lower than on other sward heights. There was no effect of sward height on wigeon’s peck rate (*F*_3,8_ = 0.894, *p* = 0.485).

There was no effect of sward height on dry matter intake rate for swan goose (ANOVA, *F*_3,8_ = 1.148, *p* = 0.387; Fig 3c) or wigeon (*F*_3,8_ = 0.941, *p* = 0.465). For bean goose, dry matter intake on 6 cm swards was significantly higher than on 1 cm swards (*F*_3,8_ = 4.878, *p* = 0.032).

Dry matter intake/peck of swan goose and bean goose were significantly different among different swards heights (ANOVA, swan goose: *F*_3,8_ = 5.944, *p* = 0.02; bean goose: *F*_3,8_ = 9.559, *p* = 0.005), as both species had a higher dry matter intake/peck when foraging on taller swards (Fig 3d). For wigeon, no significant effect was found of sward height (*F*_3,8_ = 0.318, *p* = 0.812).

Nitrogen intake rate of swan goose and wigeon was similar on different sward height (ANOVA, swan goose: *F*_3,8_ = 1.990, *p* = 0.194; wigeon: *F*_3,8_ = 3.546, *p* = 0.068; Fig 3e), but nitrogen intake rate of bean goose on 6 cm swards was significantly higher than on shorter swards (ANOVA, *F*_3,8_ = 7.424, *p* = 0.011).

A Type II functional response was the best model for the nitrogen intake rates of both bean goose (△AIC_C_ = 0.5) and wigeon (△AIC_C_ = 2.3; Table 2). A Type I functional response described the relationship between sward height and nitrogen intake rate of swan goose best (△AIC_C_ = 0; Table 2), following the rule that a simpler model should be selected when △AIC_C_ is less than 2 (Burnham and Anderson 2002), as the Type II functional response had a similar AIC-value for swan goose, but an additional model term.

**Table 2:** Comparing the functional response in relation to sward height based on nitrogen intake rate (mg/min). Type I (linear), Type II, and Type IV (quadratic). AIC_C_ values were used to decide upon the best fitting model. H = Swards height (cm).

	Wigeon					Swan goose					Bean goose				
	Model	AIC_C_	*logL*	*k*	*R*^*2*^	Model	AIC_C_	*logL*	*k*	*R*^*2*^	Model	AIC_C_	*logL*	*k*	*R*^*2*^
Type I	0.06^#^H+0.84^#^	7.0	-0.3	1	0.34	0.16^#^H+3.26^#^	29.8*	-11.7	1	0.37	0.48^#^H+2.27^#^	52.4	-23.0	1	0.44
Type II	1.63^#^H/(1+1.12H)	4.2*	1.6	2	0.50	9.45^#^H/(1+1.98H)	29.8*	-11.3	2	0.43	2.87^#^H/(1+0.34H)	50.3*	-21.5	2	0.53
Type IV	0.50^#^+0.27^#^H-0.02^#^H^2^	6.5	2.3	3	0.57	2.91^#^+0.38H-0.02H^2^	33.7	-11.3	3	0.41	-0.15+2.0^#^H-0.15^#^H^2^	50.8	-19.9	3	0.66

^#^denotes a significant coefficient (*p* < 0.05)

* denotes the best model fits, △AIC<2

### Competition experiment

The behaviour of swan goose was similar among individual birds. Peck rate of wigeon differed among individual birds when foraging on 1 and 3 cm swards height, whereas bean goose showed stronger individual differences in percentage of feeding time and peck rate (S1 Table), and hence individual was used as a random factor in our model.

The percentage of feeding time and time being vigilant of all three species was not affected by interference competition, as indicated by the model with the lowest AIC, the null model without fixed factors (Tables 3 and 4). All models, also the null models, always included all random factors (session, enclosure number, and individual).

**Table 3:** Results of the best GLMMs to test whether the percentage feeding time (arcsin transformed) was affected by sward height and competitors based on AIC. In the model sward height, competitor and their interaction term were included as fixed factors, whereas session, enclosure number, and individual were always included as random factors in all models. NS indicates that the parameter was not included in the model. All models with △AIC<2, and the next best model are reported (AIC = Akaike Information Criterion. *df*; logLik, △AIC = AIC(i)—AIC(min); ωi = Akaike weights).

Species	Model	Height (β)	Competitor (β)	Height × competitor (β)	*df*	logLik	AIC	△AIC	ωi
Bean goose	1	NS	NS	NS	5	273.2	-536.4	0.00	0.985
	2	-0.003	NS	NS	6	269.5	-527.1	9.35	0.009
Swan goose	1	NS	NS	NS	5	254.8	-499.7	0.00	0.991
	2	NS	-0.003	NS	5	250.5	-489.1	10.59	0.005
Wigeon	1	NS	NS	NS	5	169.7	-329.3	0.00	0.987
	2	NS	-0.005	NS	6	165.9	-319.7	9.59	0.008

**Table 4:** Results of the best GLMMs to test whether the percentage vigilant time (arcsin transformed) was affected by sward height and competitors. In the model sward height, competitor and their interaction term were included as fixed factors, whereas session, enclosure number, and individual were always included as random factors in all models. NS indicates that the parameter was not included in the model. All models with △AIC<2, and the next best model are reported (AIC = Akaike Information Criterion. *df*; logLik, △AIC = AIC(i)—AIC(min); ωi = Akaike weights).

Species	Model	Height (β)	Competitor (β)	Height × competitor (β)	*df*	logLik	AIC	△AIC	ωi
Bean goose	1	0.009	NS	NS	6	104.7	-197.4	0.00	0.511
	2	NS	NS	NS	5	103.6	-197.3	0.15	0.474
	3	-0.010	0.009	NS	7	101.5	-189.1	8.34	0.008
Swan goose	1	NS	NS	NS	5	327.5	-645.0	0.00	0.992
	2	0.003	NS	NS	6	323.3	-634.5	10.51	0.005
Wigeon	1	NS	NS	NS	5	130.0	-250.0	0.00	0.974
	2	-0.013	NS	NS	6	127.2	-242.4	7.54	0.022

**Fig 4:**
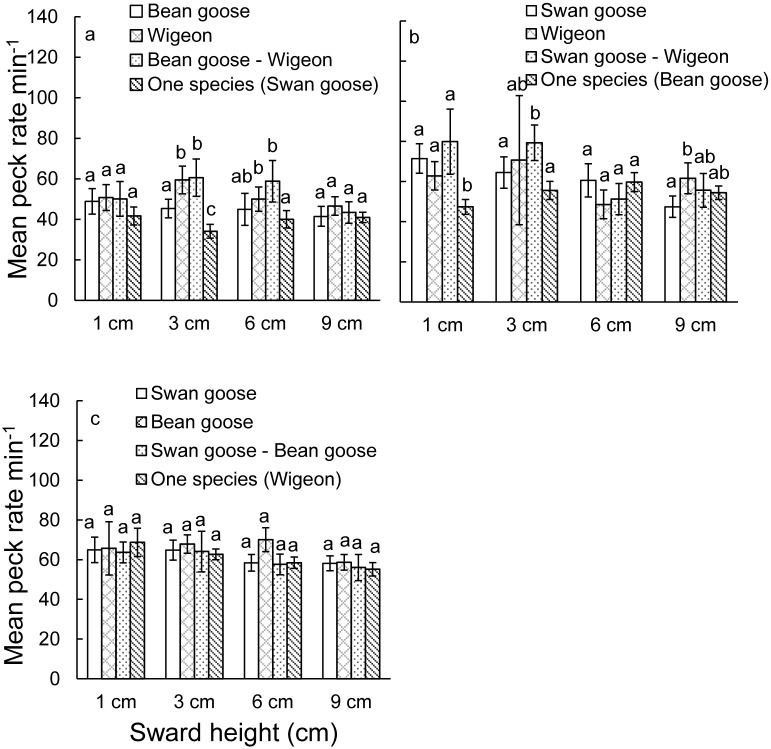
Mean peck rates (± 95% CI, untransformed data) in plots of different sward heights and with differently sized competitors a: swan goose, b: bean goose; c: wigeon. Different letters represent the results of the Tukey post-hoc test, which indicate differences between peck rate between competitors within the sward height classes.

Peck rate of all three grazing Anatidae species was affected by differently sized competitors as the competitor term was included in all best models (Table 5). Swan goose increased peck rate on 3 and 6 cm high swards when they were feeding together with other species (Fig 4a). Peck rates of bean goose on 1 and 3 cm high swards were significantly quicker when with interspecific competitors (Fig 4b), compared to the control without interspecific competitors. For wigeon, when feeding with bean goose, the peck rate on 6 cm high swards was significant quicker comparing to feed without competitors (Fig 4c).

**Table 5:** Results of the best GLMMs to test whether the peck rate was affected by sward height and competitors. In the model sward height, competitor and their interaction term were included as fixed factors, whereas session, enclosure number, and individual were always included as random factors in all models. NS indicates that the parameter was not included in the model. All models with △AIC<2, and the next best model are reported (AIC = Akaike Information Criterion. *df*; logLik, △AIC = AIC(i)—AIC(min); ωi = Akaike weights).

Species	Model	Height (β)	Competitor (β)	Height × competitor (β)	*df*	logLik	AIC	△AIC	ωi
Bean goose	1	-2.956	-1.697	0.362	8	-2246	4508	0.00	0.958
	2	NS	-1.290	NS	6	-2251	4515	6.87	0.031
Swan goose	1	NS	0.905	NS	6	-2288	4588	0.00	0.464
	2	NS	NS	NS	5	-2289	4589	0.83	0.307
	3	-0.181	0.950	NS	7	-2288	4591	2.49	0.133
Wigeon	1	-1.122	NS	NS	6	-2212	4257	0.00	0.544
	2	-1.102	-0.582	NS	7	-2122	4257	0.77	0.370
	3	-0.480	0.098	-0.132	8	-2212	4260	3.70	0.086

### Instantaneous intake rate and nitrogen intake

**Fig 5:**
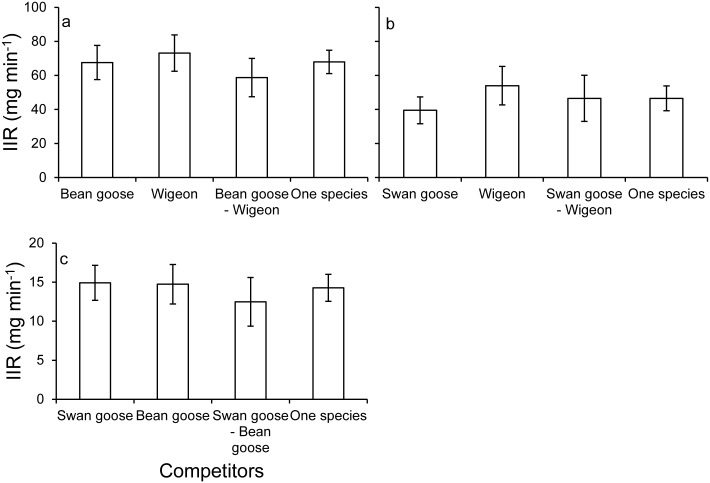
Mean instantaneous intake rate (± 95% CI) with differently sized competitors. a: swan goose, b: bean goose; c: wigeon.

**Fig 6:**
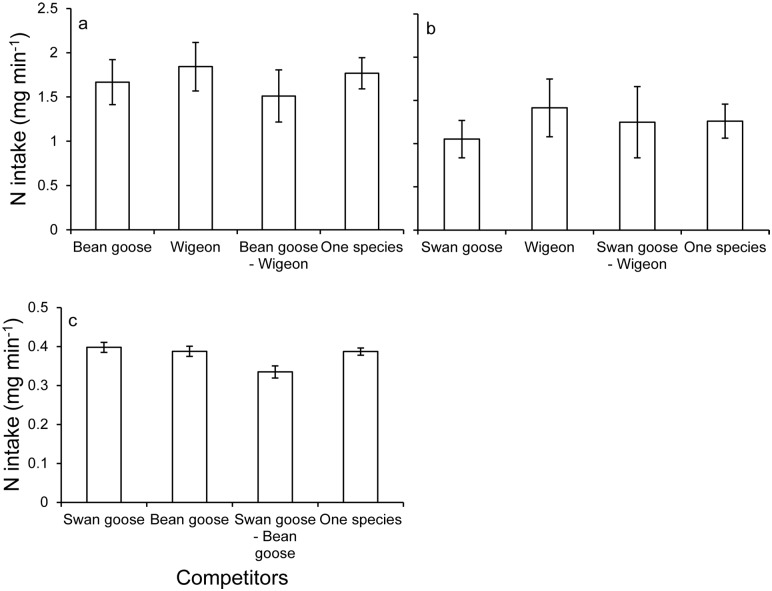
Mean nitrogen intake (± 95% CI) with differently sized competitors. a: swan goose, b: bean goose; c: wigeon. Letters represent the results of the Tukey post-hoc test, indicating differences in nitrogen intake among different species combinations.

For all species, no significant differences were found for instantaneous intake rate (ANOVA, swan goose: *F*_3,270_ = 0.791, *p* = 0.5; bean goose: *F*_3,236_ = 1.324, *p* = 0.267; wigeon: *F*_3,238_ = 0.653, *p* = 0.582; Fig 5) and nitrogen intake (ANOVA, swan goose: *F*_3,270_ = 0.762, *p* = 0.516; bean goose: *F*_3,236_ = 0.998, *p* = 0.395; wigeon: *F*_3,238_ = 0.622, *p* = 0.602; Fig 6) when feeding together with other species.

## Discussion

Our results showed that all species increased their peck rate when foraging with competitors to compensate for the loss of foraging intake when under interference competition. When they foraged with competitors, the mean percentage of feeding time and time being vigilant of all studied species remained similar (Tables 3 and 4). Comparing the peck rate with and without competitors showed that the peck rate increased under competition (Table 5, Fig 4). Both instantaneous intake rate and nitrogen intake were not significantly different over different species combinations (Figs 4 and 5), indicating that herbivorous birds can change their feeding behaviour and thereby probably compensate for a loss in intake as a results of interspecific competition. We also found that both bean goose and wigeon followed a Type II functional response, but swan goose, however, followed a Type I functional response over the range of sward heights studied (Table 2).

The nitrogen content of the vegetation is an important resource for herbivores because nitrogen is a construction element in the cell structure and it is required for all metabolic processes. Former studies often showed that the plant nitrogen content decreases with increasing plant height [1,13]. Our result, however, showed a dome-shaped relationship where the nitrogen content of the swards increased with the swards height until it reached a maximum at a sward height of 6 cm and then decreased, in line with the result of Van der Graaf et al. [17]. In *Carex* leaves, the nitrogen concentration drops from the top of the leaf to the leaf’s base [14]. Consequently and unintentionally, we might have altered the nitrogen content in our experiments by clipping the swards to the desired heights. Nevertheless, our clipping corresponded with a natural winter situation, as in this period large numbers of herbivorous Anatidae visit these sedge meadows [47,48] and the grazing pressure is very high, also by livestock [49]. Hence, herbivorous Anatidae species also modify the nitrogen distribution in sedge meadows by eating the nitrogen rich leaf tips and decreasing the sward height, as simulated in our clipping treatments.

The most common functional response in vertebrate herbivores is a Type II functional response [6]. A Type IV functional response curve, a dome shape, has been reported several times in Anatidae herbivores [5,7,8,42]. However, as shown in our results (Table 2, Fig 3e), a Type I functional response was found for swan goose and a Type II functional response yielded the best fit for both bean goose and wigeon over the range of heights studied. However, as the experiment was carried out using birds in single species flocks, these measurements do not yield interference-free functional responses. As our experiments were carried out on natural vegetation, our results may better simulate foraging intake in winter than using hand-constructed swards. With increasing sward height, peck rate of bean goose decreased (Fig 3b). However, the mean instantaneous intake rate still increased with increasing sward height (Fig 3d), which could compensate for the lower peck rate, triggering an increase in the instantaneous intake rate (Fig 3c). Moreover, the nitrogen content was higher in the taller swards (Fig 2). As above, we could explain why a Type II functional response for nitrogen intake occurs. Hence, we suggest that bean goose prefer the taller swards. Swan goose had the lowest peck rate among the three Anatidae species and peck rate decreased with increasing sward height (Fig 3b). However, the peck size of swan goose was the largest and the instantaneous intake rate was still increasing on the taller swards (Fig 3c and 3d). As shown in Table 2, a Type I functional response was found, which revealed that swan goose also preferred the taller swards. Wigeon was the smallest species in our research and we had predicted that the intake would follow a Type IV functional response. However, a Type II functional response fitted the data best (Table 2), which indicates that wigeon also preferred the tallest swards. Because of bill morphological restrictions, the peck size of wigeon was the smallest (Fig 3d). Since wigeon always maintained a high peck rate, the nitrogen intake was also still increasing with increasing sward height (Fig 3e). Taller swards might also limit their peck rate if we would have carried out this experiment on even taller swards, and we suggest that a Type IV functional response might still be found under a larger sward height gradient.

Interference competition between Anatidae species resulted in avoidance behaviour and aggressive interactions [26,32,34]. Avoidance behaviour was visible during the observations when subordinate individuals moved aside for dominant individuals of other species, whether this behaviour was costly in terms of time and energy or reduced aggressive interactions cannot be derived from our observations. However, of the 45 recorded aggressive encounters, 28 occurred between swan goose and bean goose, with swan goose as the dominant species, winning all encounters. Interspecific dominance was apparently not determined by body mass but by body height: swan goose was on average 500 g lighter than bean goose but 14 cm taller at an upright position. This result was consistent with results from a field study [50]. It might also be that body mass was affected by using tame individuals, as wild living swan goose are expected to be heavier than bean goose [51]. Intraspecific aggressive interactions were never observed.

Aggressive interactions should be costly for both subordinate and dominant species when involved in a fight or pursuit in terms of lost foraging time, energy cost and the risk of injuries [32,37,52]. Increasing the amount of time being vigilant will also increase the animals’ energetic expenditure and therefore decrease their fitness [53–55]. Hence, feeding time and/or peck rate should increase to compensate for the cost of these behavioural activities [26,30,52,56]. Indeed, we recorded the compensation behaviour for both subordinate and dominant species by increasing their peck rate. Swan goose increased their peck rate by 10–15 pecks per minute on 3 and 6 cm high swards when feeding with other competitors. The peck rate of bean goose was quicker on 1 and 3 cm high swards under interference competition. When feeding together with bean goose, wigeon increased their peck rate on 6 cm high swards. That peck rate on 9 cm high swards did not change for all studied species under competition may be explained by increasing handling time on the taller swards and hence animals faced a bottleneck effect. In result, the instantaneous intake rate and the nitrogen intake were equal over different species combinations (Figs 4 and 5), indicating that all species were able to compensate for the intake loss by this adaptive behaviour.

We failed to find large changes in feeding time allocation and time being vigilant under interference competition. The results from field studies suggest that flock size may affect animal behaviour under interference competition. For example, Mallards adjusted their vigilance behaviour in response to changes in flock size [57]. Greylag geese avoid foraging together with large flocks of Pink-footed geese [38]. In our experiment, we therefore standardized the flock size of each species to avoid this group size effect,

In conclusion, our results emphasize the importance of behavioural plasticity under interference competition. Although the mean percentage of feeding time and time being vigilant of all studied species did not change when feeding with competitors, both dominant species (swan goose) and subordinate species (bean goose and wigeon) increased their peck rate to compensate for the negative effects of competition, such as increasing aggressive behaviour and increased time being vigilant. This adaption behaviour may be of great importance for grazing Anatidae when resources are limited, increasing their survival rate during the wintering period and the breeding success afterwards.

## Supporting Information

S1 Fig Illustration of the experimental setup. (DOCX)

S1 Table Results of Kruskal-Wallis and *F* test to test if the behaviour of each species differed among different individuals. (DOCX)
