# Supplementary material for: Experimental Evidence Shows the Importance of Behavioural Plasticity and Body Size under Competition in Waterfowl
Source: PLoS One. 2016 Oct 11;11(10):e0164606. doi: 10.1371/journal.pone.0164606 (PMC5058547; doi:10.1371/journal.pone.0164606)
Supplement: S1 Fig — (DOCX) [file pone.0164606.s001.docx]

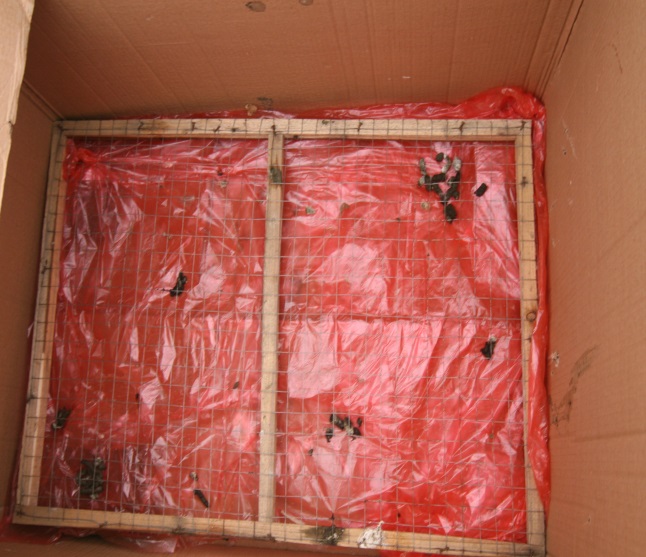

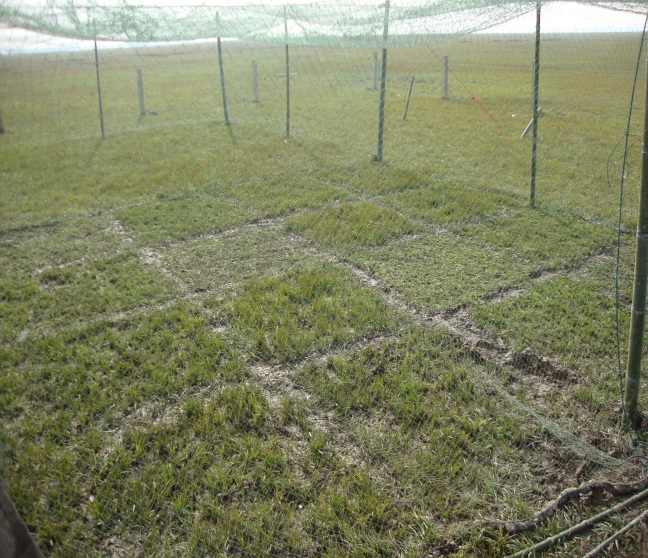

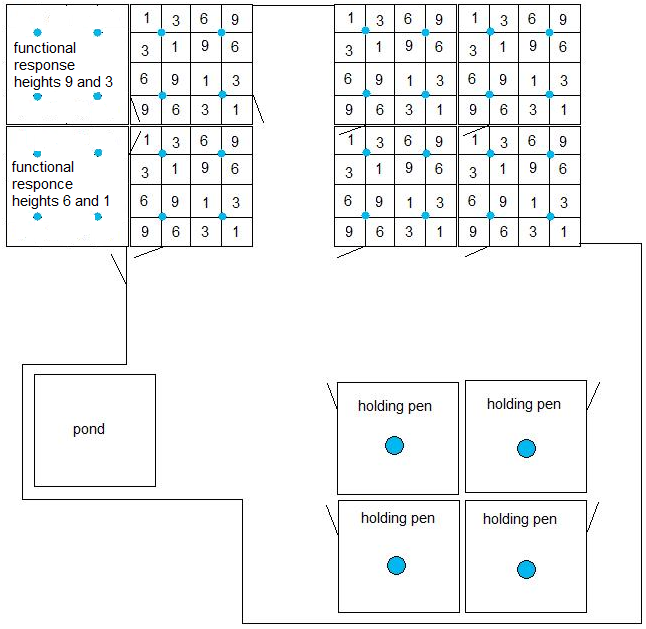

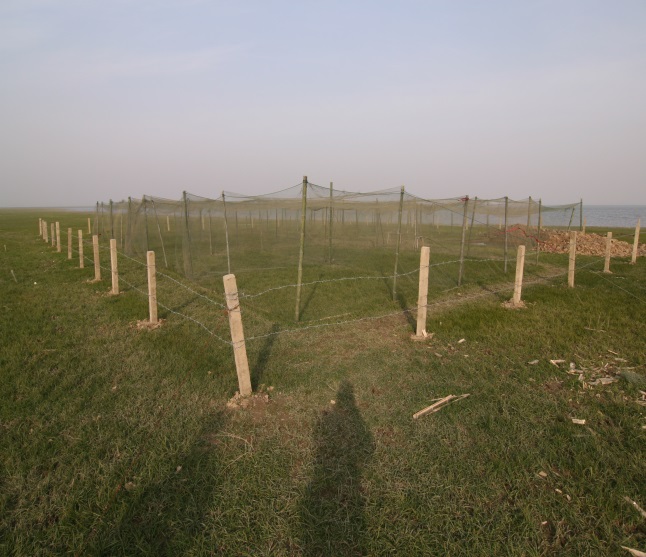


d

c

a

b

S1 Figure: Pictures illustrating the experimental setup. a: a diagram of the enclosures setup. The enclosure consisted of a pond, eight experimental enclosures (5 × 5 m, with 2 m high wire fence), and four holding pens. The blue dots (small and large) indicate the locations where clean water was available, and the oblique lines indicate the entrances. b: overview of the enclosure. c: swards were mowed to different heights (1, 3, 6, 9 cm) following a randomized Latin square design. d: container with a mesh floor used to collect the droppings of the goose after trial.
