# Supplementary material for: Experimental Evidence Shows the Importance of Behavioural Plasticity and Body Size under Competition in Waterfowl
Source: PLoS One. 2016 Oct 11;11(10):e0164606. doi: 10.1371/journal.pone.0164606 (PMC5058547; doi:10.1371/journal.pone.0164606)
Supplement: S1 Table — (DOCX) [file pone.0164606.s002.docx]

**S1 Table: Results of Kruskal-Wallis (**χ2) **and *F*-test to test if the behaviour of each species differed among different individuals. N indicates sample size.**

|  |  | Swan goose | | | | | | |  | Bean goose | | | | | | |  | Wigeon | | | | | | |
| --- | --- | --- | --- | --- | --- | --- | --- | --- | --- | --- | --- | --- | --- | --- | --- | --- | --- | --- | --- | --- | --- | --- | --- | --- |
|  |  | Percentage of feeding time | | |  | Peck rate | | |  | Percentage of feeding time | | |  | Peck rate | | |  | Percentage of feeding time | | |  | Peck rate | | |
| Swards height (cm) |  | χ2 | *P* | N |  | *F* | *p* | N |  | χ2 | *p* | N |  | *F* | *p* | N |  | χ2 | *p* | N |  | *F* | *p* | N |
| 1 |  | 4.325 | 0.504 | 65 |  | 0.992 | 0.433 | 52 |  | 17.133 | **0.004** | 52 |  | 0.271 | 0.926 | 38 |  | 7.413 | 0.192 | 20 |  | 3.063 | **0.030** | 19 |
| 3 |  | 6.763 | 0.239 | 54 |  | 0.526 | 0.754 | 32 |  | 13.399 | **0.020** | 63 |  | 6.194 | **<0.001** | 47 |  | 4.646 | 0.590 | 51 |  | 4.303 | **0.001** | 108 |
| 6 |  | 0.945 | 0.967 | 69 |  | 1.503 | 0.205 | 56 |  | 9.098 | 0.105 | 90 |  | 7.285 | **<0.001** | 69 |  | 10.072 | 0.122 | 53 |  | 1.067 | 0.392 | 73 |
| 9 |  | 6.098 | 0.297 | 80 |  | 1.424 | 0.224 | 88 |  | 3.151 | 0.677 | 113 |  | 7.381 | **<0.001** | 99 |  | 8.597 | 0.198 | 47 |  | 1.806 | 0.120 | 53 |
